# Radiographic and clinical analysis of pelvic triple osteotomy for adult hip dysplasia

**DOI:** 10.1186/1749-799X-8-17

**Published:** 2013-06-07

**Authors:** Antony R Liddell, Gareth Prosser

**Affiliations:** 1 Department of Orthopaedic Surgery, Royal Perth Hospital, Wellington St, Perth, WA 6000, Australia; 2 Department of Orthopaedic Surgery, Fremantle Hospital, Alma St, Fremantle, WA 6060, Australia

**Keywords:** Adult hip dysplasia, Triple osteotomy, Hip^2^Norm

## Abstract

**Background:**

Adult Hip Dysplasia (AHD) has been strongly linked with the development of hip osteoarthritis. The complexity and therefore resultant steep learning curve of the Bernese osteotomy for AHD has been well described. The purpose of this study was to analyse the efficacy of a technically less demanding interlocking pelvic triple osteotomy.

**Methods:**

Pre and postoperative pelvic radiographs of 8 hips in 7 patients who underwent pelvic osteotomy between January 2010 and December 2011 were corrected to a standardised orientation using a validated software package, Hip^2^Norm^TM^, and this tool was then used to measure hip parameters used for assessing dysplasia. The Lateral Centre Edge Angle (LCEA), the Acetabular Index of the Weight-Bearing Zone (AIWB), and the percentage Acetabular Coverage of the Femoral Head (ACFH) were all calculated and compared. Oxford hip scores, WOMAC hip scores, and UCLA activity scores were clinical outcome measures.

**Results:**

Average LCEA correction was 23.8 deg, from a mean of 8.8 deg preoperatively to 32.6 deg postoperatively. AIWB was corrected an average of 21.3 deg (mean 22.5 to 1.2 deg postoperatively) and ACFH was increased on average 23.8% (mean 59.0 to 82.8% postoperatively). At a minimum follow-up of 3 months Oxford hip scores improved from an average of 19.6 preoperatively to 39.4, and the mean UCLA activity index was increased from 3.3 to 7.1 postoperatively. There were two technical complications in the studied procedures, which have resulted in no long-term sequelae.

**Conclusions:**

This study demonstrates the safe and effective use of an interlocking pelvic triple osteotomy to provide correction of radiological parameters and symptomatic improvement of AHD.

## Background

Residual developmental dysplasia of the adult acetabulum (AHD) is strongly associated with secondary arthritis of the hip joint [1]. The dysplastic acetabulum is predisposed to arthritic change for several biomechanical reasons [2]. Re-directional pelvic osteotomies have been developed to improve the biomechanical properties of the dysplastic acetabulum, reduce symptomatic joint dysfunction, and delay or even avert the development of arthritis [3,4]. The Bernese periacetabular osteotomy as developed by Ganz has become popularly utilised for this indication [3,5]. Whilst providing for a powerful correction of acetabular position, this osteotomy is technically demanding, and is acknowledged to have a steep learning curve [5,6]. This study details the use of an interlocking version of the Tönnis osteotomy [7].

**Figure 1:**
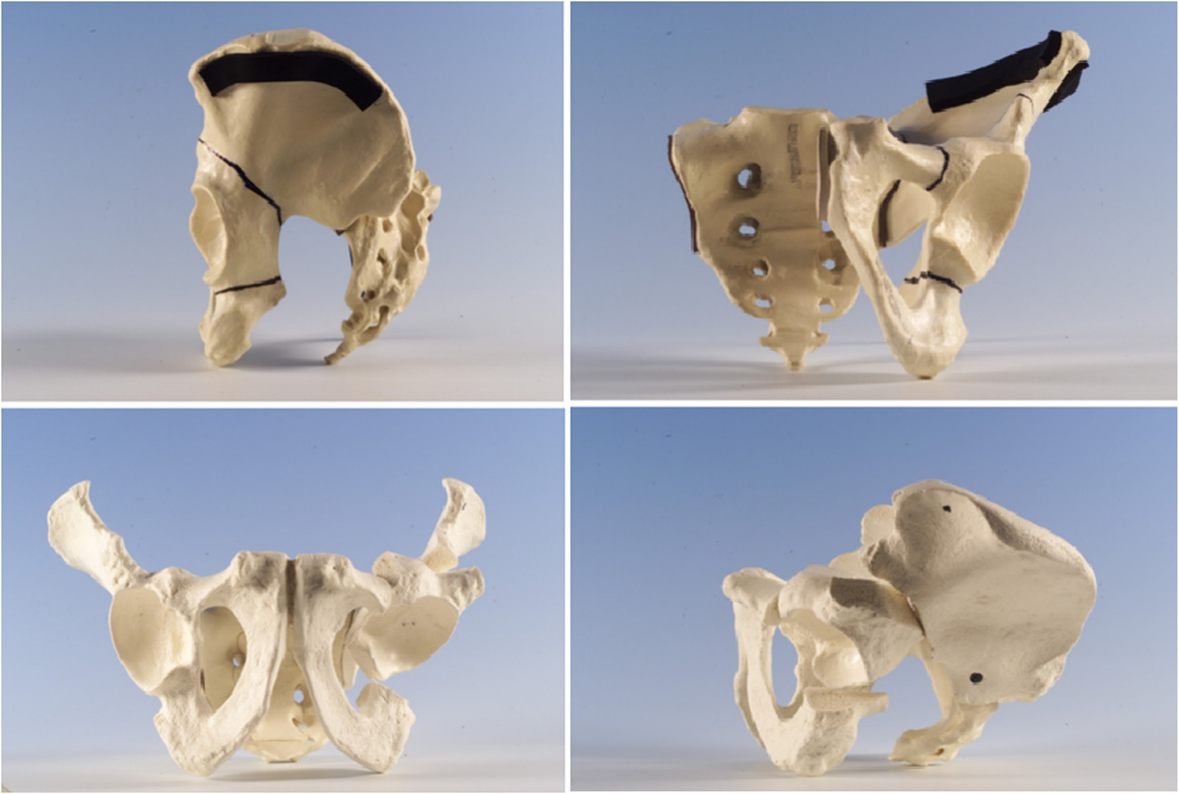
Three dimensional representation of Interlocking Pelvic Triple Osteotomy - preoperative cuts and postoperative orientation.

**Figure 2:**
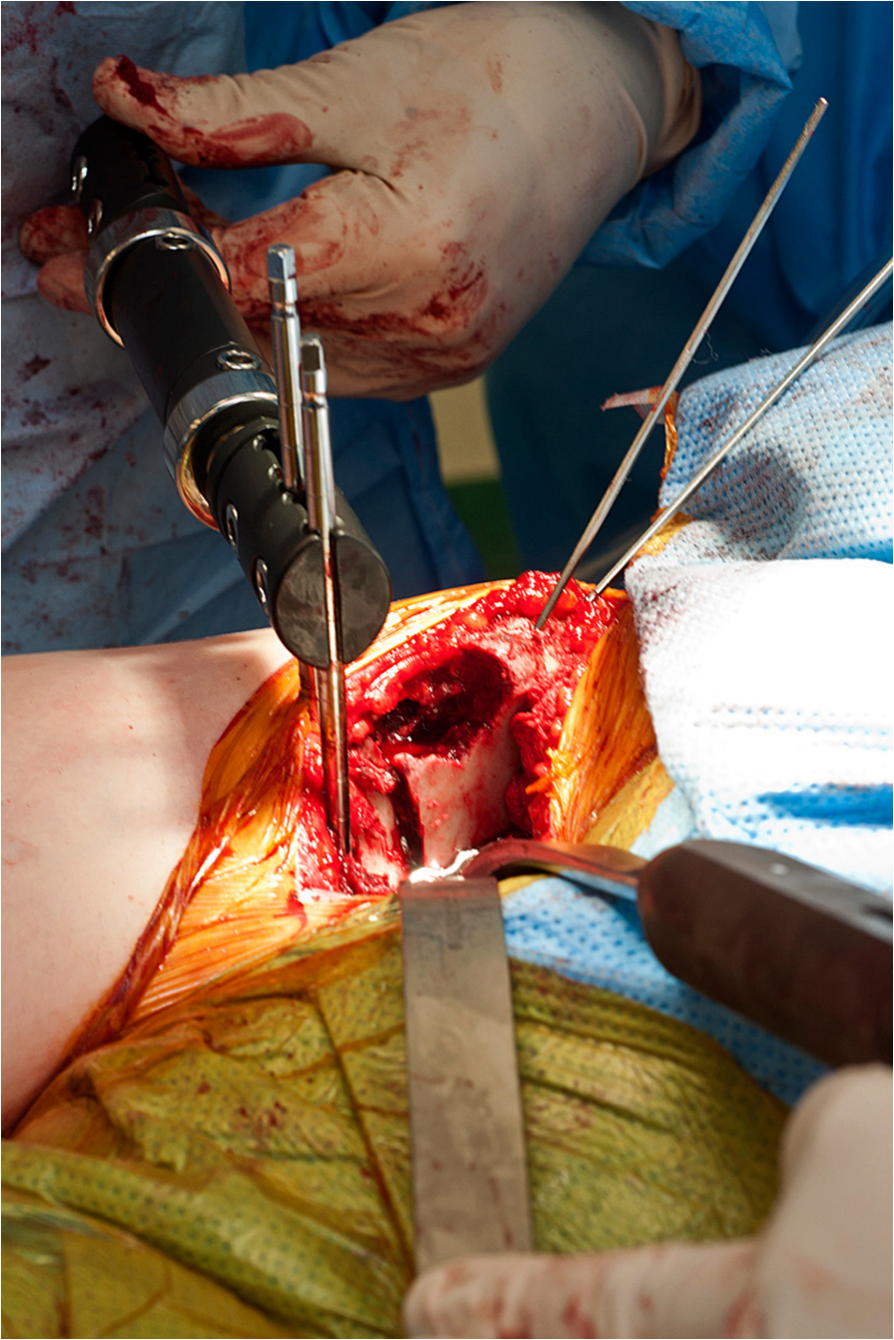
Clinical photograph demonstrating use of the external fixator to control orientation of the osteotomy.

**Figure 3:**
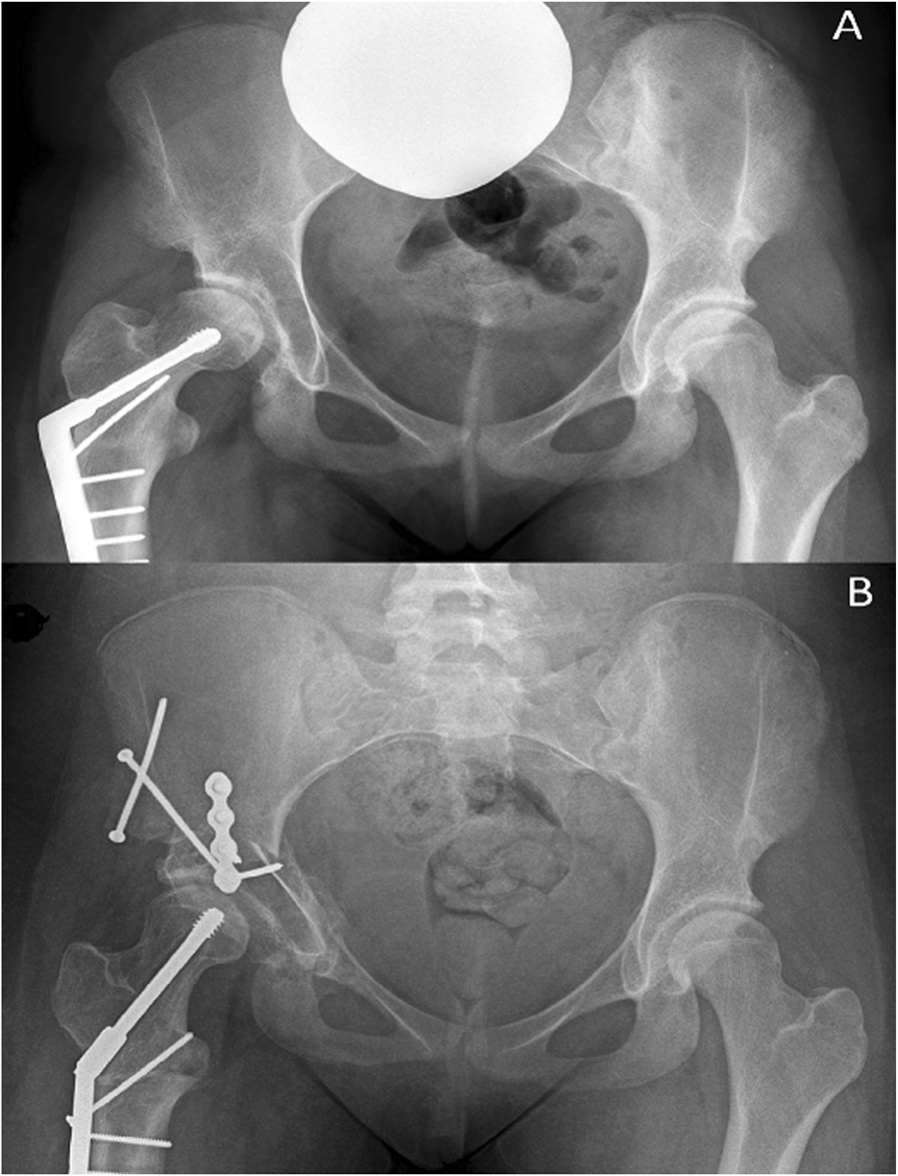
**Example of preoperative (A) and postoperative (B) radiographs following Interlocking Pelvic Triple Osteotomy.** Note in the preoperative film evidence of a previous proximal femoral varus osteotomy. In the postoperative film, the acetabular orientation has been corrected, and the proximal femur has been repositioned to a physiological orientation.

**Table 1:** Patient ages, & pre and postoperative clinical outcome measures

**Name (Side)**	**Age at surgery**	**Preop. Oxford**	**Postop. Oxford**	**Preop. UCLA**	**Postop. UCLA**
Patient 1 (Lt)	32	7	39	2	7
Patient 2 (Rt)	25	25	42	4	7
Patient 2 (Lt)	25	42	44	7	10
Patient 3 (Rt)	26	28	40	4	6
Patient 4 (Rt)	16	9	44	2	7
Patient 5 (Lt)	21	29	40	5	9
Patient 6 (Rt)	17	15	33	3	5
Patient 7 (Rt)	22	24	32	4	6
**Mean**	**23**	**22.4**	**39.3**	**3.9**	**7.1**

**Table 2:** Pre and postoperative radiological indices

**Name (Side)**	**Preop. LCEA**	**Postop. LCEA**	**Preop. AIWB**	**Postop. AIWB**	**Preop. ACFH**	**Postop. ACFH**
Patient 1 (Lt)	3	34	32	2	57	89.2
Patient 2 (Rt)	7	43	22	2	53.2	89
Patient 2 (Lt)	3	27	21	0	60.1	84.6
Patient 3 (Rt)	9	29	22	6	62.6	81.6
Patient 4 (Rt)	12	24	20	0	57.3	74.9
Patient 5 (Lt)	12	29	17	0	65	80.1
Patient 6 (Rt)	10	45	25	0	57.9	80.4
Patient 7 (Rt)	14	29	21	0	61	82
**Mean**	**8.8**	**32.5**	**22.5**	**1.3**	**59.3**	**82.7**

**Figure 4:**
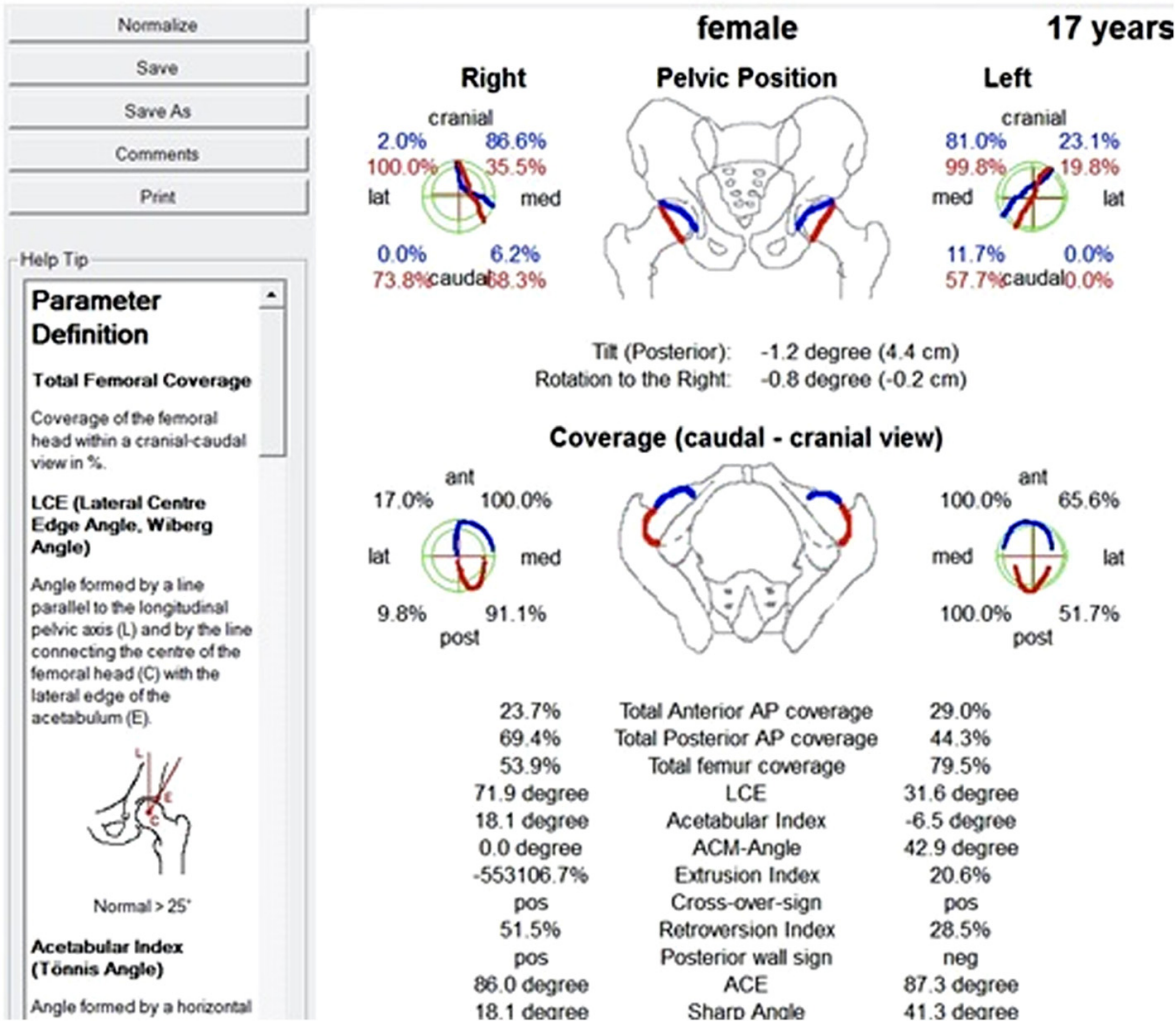
**Screen shot of Hip **^**2 **^**Norm**^**TM **^**software applied to post operative radiograph of study patient.** Note the calculation of Acetabular Coverage of the Femoral Head.

With any surgery around the acetabulum accurate pre and postoperative radiological assessment is imperative to quantify the magnitude and direction of correction that is desired and subsequently obtained. Many indices have been described for the radiological evaluation of hip dysplasia. These include Wiburg’s Lateral Centre Edge Angle (LCEA), the Acetabular Index of the Weight-Bearing Zone (AIWB) of Tönnis, and the Femoral Head Extrusion Index (FHEI) [1,8]. Common to all of these indices is the critical influence of spatial pelvic orientation at the time of radiograph capture [8,9]. This variability can significantly affect the ability to reliably compare studies either between different patients or studies performed at different times in the same patient. As a result of the dysplastic acetabular morphology, the percentage Acetabular Coverage of the Femoral Head (ACFH) has been shown to be decreased in AHD. This value is most accurately calculated using CT scans, and a CT-based study has found average values for ACFH to be 73% and 51% in normal and dysplastic hips respectively [10].

Attempts to standardise the pelvic orientation for radiological assessment have lead to the development of a validated computer software tool, Hip^2^Norm^™ ^, by a group from the University of Bern [9]. The tool is applied to the digitised radiographic images, and used to calculate the virtual three-dimensional morphology of the acetabulum with reference to the pelvis. After plotting easily identifiable points on the AP pelvic radiograph, the Hip^2^Norm software program re-orientates the pelvis to a standardised neutral position. From here radiographic hip parameters are calculated in a reproducible way, uninfluenced by the variations of pelvic position relative to the xray beam [9].

The purpose of this study was to assess the effectiveness of the interlocking pelvic triple osteotomy in achieving both radiological correction of Adult Hip Dysplasia, as well as symptomatic improvement postoperatively. Secondarily, the use of the computerised normalisation of pelvic orientation prior to radiological scoring is demonstrated.

## Methods

All patients with a radiographic hip dysplasia who underwent an interlocking pelvic osteotomy in our hospital between 01/2010 and 11/2011 were included in the present study. These are the first eight pelvic osteotomies performed by the senior author. All data were collected according to the terms of reference specified by the hospital clinical governance unit as directed by the regional ethics committee.

Criteria for consideration of pelvic osteotomy were employed. The patient had to exhibit symptomatic hip dysplasia, and be aged below 35 years. Additionally, the patient had to have little or no radiographic evidence of arthritis, and a closed triradiate cartilage. To ensure efficacy of treatment, adequate, concentric joint reduction had to be demonstrated on a hip radiograph with the femur abducted and internally rotated (AIR view).

The pelvic osteotomy was performed as developed by J O’Hara and described completely by Kumar et al. in 2002 [7]. A two incision approach is utilised. Initially the patient is placed in the lateral position, and after superficial dissection, mobilisation of the sciatic nerve, and elevation of the short external rotators, an ischial osteotomy through the sub-cotyloid groove is performed from the greater sciatic notch to the obturator foramen. Following confirmation of completion of the osteotomy, the wound is closed and the patient turned supine. An anterior, bikini incision is performed, and an anterior superior iliac spine osteotomy performed. Iliacus is mobilised off the inner table, and osteotomy of the superior pubic ramus is then performed close to the acetabulum. An external fixator is applied with two 5 mm pins are placed in the acetabular fragment to act as an aid to mobilisation and an indicator of correction. A three sided iliac cut is performed with a reciprocating saw. The acuity of the two angles formed between the three limbs of the osteotomy determines the magnitude of correction achieved. The angles are made equal, to allow interlocking of the rotated fragment (Figure 1). The osteotomy is held in position with a plate, and the fixator pins removed [7] (Figure 2). This technique allows improvement of lateral coverage, correction of anteversion, and medialisation of centre of rotation (Figure 3).

Digitised pre and postoperative pelvic radiographs were assessed using the Hip^2^Norm^™^ software package. Using the computer mouse, several anatomic landmarks were mapped manually. These include the inferior margins of the teardrops for horizontal referencing, the femoral head centres and the projection of the anterior and posterior acetabular rims. After virtual 3-dimensional reorientation, calculation of three established hip indices is performed.

The LCEA is obtained by measuring the angle formed by the intersection of a vertical line through the centre of the femoral head and a line extending through the centre of the femoral head and the lateral margin of the femoral sourcil. Most authors define dysplasia as a LCEA of less than 20°, with borderline dysplasia measuring between 20 and 25°. The AIWB is obtained by measuring the angle formed by the intersection of the horizontal line and a line drawn between the medial and lateral extent of the superior weight-bearing zone. Normal values for the AIWB are less than 10 degrees. The software calculation of the ACFH is well described in the article by Tannast et al. The calculation is based on a spherical model of the hip, and correction is again made for pelvic position and a conical xray beam [9] (Figure 4).

Pre and three-month postoperative functional and symptomatic hip scoring was performed using a combination of two validated instruments [11]. The joint specific Oxford Hip Score (OHS) is a patient perspective outcome measure assessing pain and functional ability [12]. 12 questions are scored from 0 to 4 giving a best outcome of 48. The University of California, Los Angeles (UCLA) activity scale is a 10 point scale which provides a reliable assessment of patient activity level [13,14].

The level for rejection of the null hypothesis was set at 0.05 (α = 0.05) for all comparison tests in this study. Paired *t* tests were performed to compare the numeric variables between pre-operative and post-operative visits.

## Results

A total of 8 hips in 7 patients met the inclusion criteria within the specified period. No patients have been lost to follow-up at the time of writing. There were two complications encountered in the patient cohort. The first of these patients had a femoral nerve palsy which resolved completely after a period of 4 months. In a second patient, a nutrient vessel of the iliacus muscle was encountered, and after resuscitation the patient became coagulopathic. The procedure was abandoned, and she subsequently had it completed uneventfully 5 days later. Significantly, there were no cases of malpositioning, no cases of crack propagation into the acetabulum, and no cases of non-union. Mean preoperative LCEA was found to be 8.8 deg (± 3.1 deg, range 3–14 deg), all well within the accepted values for dysplasia. Postoperatively an average correction of 23.8 deg was obtained, to a mean of 32.5 deg (± 7.6 deg, range 24–45 deg), within normal range (p < 0.001). The preoperative AIWB was found to be 22.5 deg (± 4.4 deg, range 17 – 32 deg). Following an average correction of 21.3 deg, this value was 1.3 deg (± 2.1 deg, range 0–6 deg) (p < 0.001), again demonstrating normalisation of radiological indices of dysplasia. With the application of the Hip^2^Norm software, pre-operative Acetabular Coverage of the Femoral Head was calculated at 59.3% (± 3.7%, range 53.2 – 65.0%). This was corrected to 82.7% (± 4.8%, range 74.9 – 89.2%) postoperatively, an average correction of 23.4% (p < 0.001). Values for each patient are shown in Table 1.

At a minimum follow-up of 3 months clinical outcome measure improvements were found. Mean Oxford Hip Scores increased from 22.4 (± 11.6, range 7– 42) preoperatively to 39.3 (± 4.6, range 32 – 44) postoperatively (p < 0.005). UCLA activity scale scores were increased from a mean preoperative value of 3.9 (± 1.6, range 2–7) to an average postoperative value of 7.1 (± 1.6, range 7–10) (p < 0.001). Individual pre and postoperative scores as well as patient ages are shown in Table 2.

## Discussion

In the hip, up to 70% of radiographic hip osteoarthritis has been related to malformation [15]. Of the hip malformations, residual hip dysplasia has been linked with OA since Wiberg published his thesis on the subject in 1939. Whilst Wiberg found a linear relationship between the degree of radiographic dysplasia and the age of onset of OA, this finding has not been born out in modern literature [16]. Numerous studies have, however, confirmed a link between dysplasia and the presence of both radiographic and symptomatic hip OA [16,17]. Cross sectional population analysis has shown significant relationships between all frequently used radiographic indices of dysplasia and the presence of OA [17]. Both MRI and arthroscopic studies have demonstrated significant relationships between acetabular dysplasia and cartilage degeneration [18,19].

The shallow dysplastic hip is predisposed to OA for several biomechanical reasons. Obliquity of the acetabulum generates shear forces at the joint, resulting in lateralisation of the femoral head. This in turn decreases the femoral head weight-bearing area; and causes labral hypertrophy and overload [1,5]. The decrease in weight-bearing surface area increases chondral load, and as a result of lateralisation of the femoral centre of rotation, joint reaction forces (JRF) at the chondral surface are significantly increased [1,18].

Surgical attempts to improve the biomechanics of the dysplastic acetabulum have resulted in the evolution of a number of proximal femoral and pelvic osteotomies. Redirectional pelvic osteotomies have become the mainstay of surgical management of the young adult with residual hip dysplasia [5,20,21]. Steel’s triple pelvic osteotomy, described in 1965, incorporated pubic, ilial, and ischial osteotomies, therefore allowing redirection of the whole acetabulum relative to the pelvis. This was further refined by Tönnis in 1981 with a juxtaarticular osteotomy avoiding the sacro-pelvic ligaments [22]. In 1988 Ganz published the results of a single incision periacetabular osteotomy (PAO). This technique does not require complete division of the posterior column of the hemipelvis, allowing early weight-bearing and minimising changes to the shape of the true pelvis.

Whilst the Ganz osteotomy is widely regarded as the most anatomical of the redirectional acetabular osteotomies, it is acknowledged as both complex and having a steep learning curve [2,6,23]. In a 10 year review of Ganz’ first 75 PAO for dysplasia, it was noted that 8 of the first 18 cases had a major complication [24]. In a further analysis of Ganz’ first 508 POA published in 1999, it was noted that 85% of the technical complications occurred during the initial 50 procedures [25]. These observations have been confirmed in a number of series published on the technique by other authors [21,26-28]. Once through the early post operative period, acetabular osteotomies are tolerated well, with two medium term series showing survivorships and improved hip scores in greater than 80% of patients at 11.3 and 9.2 years respectively [24,29].

The current study utilises an interlocking version of the Tonnis osteotomy with is technically less demanding than the Ganz PAO. The technique was described by Kumar and O’Hara in 2002 for the management of severe Legg-Calve-Perthes Disease in a paediatric population [7]. The two incision approach provides for a stable and powerful acetabular correction with an apparent lower risk of crack propagation into the acetabulum than that reported with the Ganz series. In our experience, the use of the external fixator to guide rotation, as well as accurate preoperative planning ensured radiological correction of deformity in all cases. Our confidence in the accuracy of pelvic index measurement both pre and postoperatively was significantly increased with the utilisation of the Hip^2^Norm package. Similar to the Ganz osteotomy, the interlocking nature of the osteotomy ensured postoperative stability and allowed for early weight-bearing [7].

## Conclusions

This small consecutive series covering the first cases of the principal operating surgeons practice has demonstrated the safety and efficacy of the interlocking pelvic triple osteotomy in the management of Adult Hip Dysplasia. Radiographic correction of dysplasia was achieved in all patients in a predictable and consistent fashion. Significant symptomatic and functional improvements were identified with all instruments used. These results were obtained with few perioperative and no long-term complications.

## Competing interests

The authors declare they have no competing interests.

## Authors’ contributions

ARL and GHP designed the study, and collected and performed the analysis of the data. ARL wrote the manuscript. GHP was the senior surgeon, performing all of the osteotomies discussed. Both authors read and approved the final manuscript.
